# Association of midlife hearing impairment and hearing aid use with incident dementia: analysis of two UK-based longitudinal cohort studies

**DOI:** 10.1038/s43587-025-00914-1

**Published:** 2025-07-01

**Authors:** Marcos D. Machado-Fragua, Aurore Fayosse, Julien Dumurgier, Mika Kivimaki, Céline Ben Hassen, Gill Livingston, Claire Paquet, Séverine Sabia, Archana Singh-Manoux

**Affiliations:** 1 Université Paris Cité, Inserm U1153, Epidemiology of Ageing and Neurodegenerative Diseases, Paris, France; 2 Cognitive Neurology Center, Saint Louis—Lariboisiere—Fernand Widal Hospital, AP-HP, Université Paris Cité, Paris, France; 3 Faculty of Brain Sciences, University College London, London, UK

**Keywords:** Dementia, Medical research, Ageing

## Abstract

The prevalence of hearing impairment and dementia increases with age but the nature of their association remains unclear. Using data from two large UK-based cohort studies, we examined the longitudinal association of ‘midlife’ hearing impairment and hearing aid use with incident dementia. We found modest associations and population attributable fractions for reported and measured ‘midlife’ hearing impairment, highlighting the need to consider age at measurement of risk factors in studies that target dementia prevention.

## Abstract (web summary)

Machado-Fragua et al. report modest associations and population attributable fractions for midlife hearing impairment with dementia, highlighting the need to consider age at measurement of risk factors in studies that target dementia prevention.

## Main

Dementia affects more than 56 million people worldwide^1^, and it has recently been suggested that hearing impairment is a potentially modifiable risk factor for dementia^2–5^. Hearing loss is common at older ages^6^ and has been reported to be associated with risk of dementia^7–12^. The use of population attributable fractions (PAF) in recent meta-analyses on hearing impairment in adults (weighted PAF 7.2%, using reported hearing impairment)^3^ and ‘midlife’ hearing impairment (weighted PAF 7%, using objective measures)^2^ assumes a ‘causal’ association with dementia. Findings from studies on the use of hearing-restorative devices, hypothesized to be protective, are inconsistent; some suggest higher risk in hearing aid users^13^, some a bi-directional association^14^, some a protective effect^15,16^ and trials show no effect^17,18^.

Most dementias have a long preclinical phase, with pathophysiological changes commencing 15–20 years before clinical symptoms^19^. Findings from studies with a longer follow-up—when the exposure is measured in midlife—are likely to be informative on potential targets for primary prevention of late-onset dementia.

The few studies that used objectively measured hearing impairment in midlife (<60 years) reported a modest association with dementia^13,20,21^. In this context, our aim was to examine the association of hearing impairment and hearing aid use in ‘midlife’ with incident late-onset dementia (after 65 years) using data from the Whitehall II (WII) and UK Biobank (UKB) studies.

**Extended Data Fig. 1:**
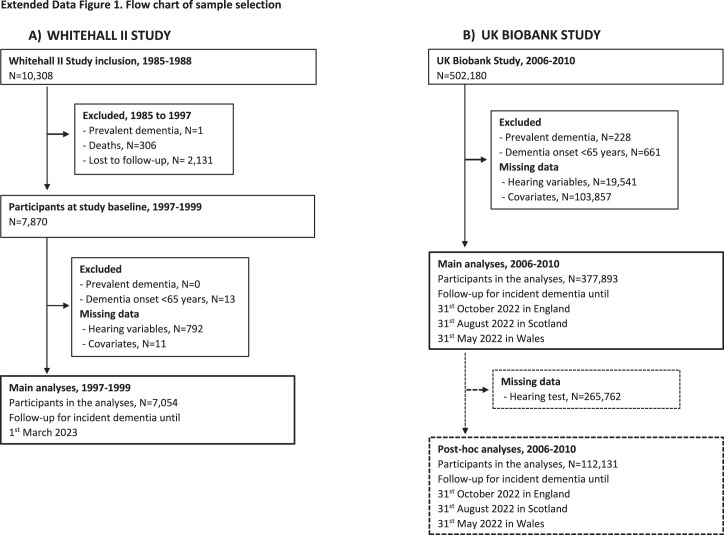
Flow chart of sample selection. a,b, Sample selection processes for the Whitehall II study (a) and for the UK Biobank study (b). The figure details the number of participants excluded and the reasons for exclusion at each stage.

Extended Data Fig. 1 shows the flow chart of sample selection in both cohorts. WII included 7,054 participants (mean age ± s.d. 55.9 ± 6.0 years; 29.5% women) with 692 incident dementia cases over a median (interquartile range (IQR)) follow-up of 24.8 (23.0 to 25.1) years. UKB included 377,893 participants (mean age ± s.d. 56.3 ± 8.1 years; 52.5% women) with 6,924, 2,814 and 1,368 incident cases of all-cause dementia, Alzheimer’s disease and vascular dementia, respectively, over a median (IQR) follow-up of 13.6 (12.9 to 14.3) years. Participants’ characteristics are shown in Table 1. The prevalence of hearing impairment was 20.5% in WII and 41.9% in UKB; hearing aid prevalence in the two studies was 2.4% and 2.9%, respectively.

**Table 1:** Characteristics of participants at baseline in the WII (1997–1999) and UKB (2006–2010) studies

	WII						UKB					
	Hearing impairment			Hearing aid use			Hearing impairment			Hearing aid use		
	No.	Yes	*P*	No.	Yes	*P*	No.	Yes	*P*	No.	Yes	*P*
*n*	5,608 (79.5)	1,446 (20.5)		6,888 (97.6)	166 (2.4)		219,599 (58.1)	158,294 (41.9)		366,898 (97.1)	10,995 (2.9)	
Age, mean (s.d.)	55.7 (6.0)	56.7 (6.1)	<0.001	55.8 (6.0)	59.7 (5.6)	<0.001	55.3 (8.2)	57.6 (7.8)	<0.001	56.1 (8.1)	61.9 (6.3)	<0.001
Women	1,708 (30.5)	373 (25.8)	0.001	2,030 (29.5)	51 (30.7)	0.73	126,279 (57.5)	71,964 (45.5)	<0.001	193,502 (52.7)	4,741 (43.1)	<0.001
Minority ethnic group	429 (7.6)	139 (9.6)	0.01	558 (8.1)	10 (6.0)	0.33	11,246 (5.1)	6,461 (4.1)	<0.001	17,396 (4.7)	311 (2.8)	<0.001
Single, widowed or divorced^a^	1,363 (24.3)	314 (21.7)	0.04	1,634 (23.7)	43 (25.9)	0.51	40,145 (18.3)	28,653 (18.1)	0.16	66,762 (18.2)	2,036 (18.5)	0.39
Low education	2,513 (44.8)	675 (46.7)	0.44	3,096 (44.9)	92 (55.4)	0.02	28,131 (12.8)	26,656 (16.8)	<0.001	51,859 (14.1)	2,928 (26.6)	<0.001
BMI ≥30 kg m^−^^2^	773 (13.8)	214 (14.8)	0.17	955 (13.9)	32 (19.3)	0.17	49,169 (22.4)	39,075 (24.7)	<0.001	85,178 (23.2)	3,066 (27.9)	<0.001
Alcohol consumption												
No consumption	915 (16.3)	239 (16.5)	0.56	1,131 (16.4)	23 (13.9)	0.44	62,580 (28.5)	46,896 (29.6)	<0.001	105,860 (28.8)	3,616 (32.9)	<0.001
1–14 units per week	2,828 (50.4)	707 (48.9)		3,444 (50.0)	91 (54.8)		80,536 (36.7)	58,064 (36.7)		134,519 (36.7)	4,081 (37.1)	
>14 units per week	1,865 (33.3)	500 (34.6)		2,313 (33.6)	52 (31.3)		76,483 (34.8)	53,334 (33.7)		126,519 (34.5)	3,298 (30.0)	
Current smoker	590 (10.5)	148 (10.2)	0.04	720 (10.5)	18 (10.8)	0.37	21,685 (9.9)	16,337 (10.3)	<0.001	37,030 (10.1)	992 (9.0)	<0.001
Poor diet^b^	1,438 (25.6)	453 (31.3)	<0.001	1,856 (26.9)	35 (21.1)	0.07	7,132 (3.2)	6,073 (3.8)	<0.001	12,861 (3.5)	344 (3.1)	0.02
PA (h per week), mean (s.d.)^c^	3.32 (3.20)	3.34 (3.20)	0.78	3.3 (3.2)	3.8 (3.9)	0.04	2,662 (2,665)	2,625 (2,743)	<0.001	2,646 (2,697)	2,649 (2,745)	0.93
Prevalent chronic disease												
Coronary heart disease	328 (5.8)	129 (8.9)	<0.001	441 (6.4)	16 (9.6)	0.09	7,668 (3.5)	9,096 (5.7)	<0.001	15,653 (4.3)	1,111 (10.1)	<0.001
Stroke	12 (0.2)	7 (0.5)	0.08	19 (0.3)	0 (0.0)	0.50	1,194 (0.5)	1,345 (0.8)	<0.001	2,370 (0.6)	169 (1.5)	<0.001
Heart failure	2 (0.04)	3 (0.2)	0.03	5 (0.1)	0 (0.0)	0.73	879 (0.4)	980 (0.6)	<0.001	1,734 (0.5)	125 (1.1)	<0.001
Hypertension	2,091 (37.3)	538 (37.2)	0.96	2,547 (37.0)	82 (49.4)	0.001	112,375 (51.2)	88,302 (55.8)	<0.001	193,412 (52.7)	7,265 (66.1)	<0.001
Diabetes	253 (4.5)	75 (5.2)	0.28	320 (4.6)	8 (4.8)	0.92	9,951 (4.5)	9,558 (6.0)	<0.001	18,444 (5.0)	1,065 (9.7)	<0.001
Cancer	135 (2.4)	30 (2.1)	0.46	161 (2.3)	4 (2.4)	0.95	11,002 (5.0)	8,678 (5.5)	<0.001	18,919 (5.2)	761 (6.9)	<0.001
Chronic kidney disease	1 (0.02)	0 (0.0)	0.61	1 (0.01)	0 (0.0)	0.88	2,178 (1.0)	2,035 (1.3)	<0.001	3,958 (1.1)	255 (2.3)	<0.001
COPD	3 (0.05)	0 (0.0)	0.38	3 (0.04)	0 (0.0)	0.79	1,906 (0.9)	2,562 (1.6)	<0.001	4,107 (1.1)	361 (3.3)	<0.001
Liver disease	2 (0.04)	1 (0.07)	0.58	3 (0.04)	0 (0.0)	0.79	1,449 (0.7)	1,291 (0.8)	<0.001	2,623 (0.7)	117 (1.1)	<0.001
Depression	271 (4.8)	96 (6.6)	0.01	356 (5.2)	11 (6.6)	0.40	17,562 (8.0)	17,866 (11.3)	<0.001	33,966 (9.3)	1,462 (13.3)	<0.001
Mental disorders	3 (0.05)	0 (0.0)	0.38	3 (0.04)	0 (0.0)	0.79	10,437 (4.8)	9,381 (5.9)	<0.001	19,125 (5.2)	693 (6.3)	<0.001
Arthritis or RA	15 (0.3)	2 (0.1)	0.37	16 (0.2)	1 (0.6)	0.34	45,008 (20.5)	40,119 (25.3)	<0.001	81,257 (22.1)	3,870 (35.2)	<0.001
Median (IQR) follow-up (years)	24.9 (23.4–25.1)	24.8 (21.4–25.1)	0.001	24.8 (23.1–25.1)	24.8 (18.8–25.1)	0.03	13.7 (12.9–14.3)	13.6 (12.8–14.3)	<0.001	13.6 (12.9–14.3)	13.5 (12.6–14.2)	<0.001
Incident dementia	519 (9.3)	173 (12.0)	0.002	666 (9.7)	26 (15.7)	0.01	3,190 (1.5)	3,734 (2.4)	<0.001	6,399 (1.7)	525 (4.8)	<0.001

Data are *n* (%), unless otherwise specified. COPD, chronic obstructive pulmonary disease; PA, physical activity; RA, rheumatoid arthritis.

^a^Living alone in the UKB.

^b^Poor diet defined by consumption of fruit and vegetables less than daily.

^c^MET (min per week) in the UKB, mean (s.d.).

Hearing impairment was assessed at ~56 years in both studies, but follow-up was longer in WII (25 years versus 14 years), and median age at dementia diagnosis was 80 years in WII, close to the mean age of dementia diagnosis in the UK, and 75 years in UKB. Adjusted for all covariates, UKB participants with hearing impairment had a higher risk of dementia (hazard ratio (HR) 1.12; 95% confidence interval (CI) 1.07, 1.17) (Table 2). This association was not statistically significant in WII (HR 1.14; 95% CI 0.96, 1.35), although HR estimates in both studies were similar. Hearing aid use was associated with incident dementia in UKB (HR 1.23; 95% CI 1.12, 1.34) but not in WII (HR 1.05; 95% CI 0.71, 1.55); the pooled estimate was 1.22 (95% CI 1.12, 1.33).

**Table 2:** Associations of reported hearing impairment and hearing aid use with incident dementia in the WII and UKB cohort studies

	No. of dementia cases (total)	Rate per 1,000 person-years (95% CI)	HR (95% CI)		
			Model 1^a^	Model 2^b^	Model 3^c^
WII^d,e^					
Hearing impairment					
No	519 (5,608)	4.09 (3.75, 4.46)	Reference	Reference	Reference
Yes	173 (1,446)	5.41 (4.66, 6.28)	1.15 (0.97, 1.37)	1.15 (0.96, 1.36)	1.14 (0.96, 1.35)
Hearing aid use					
No	666 (6,888)	4.29 (3.97, 4.63)	Reference	Reference	Reference
Yes	26 (166)	7.22 (4.92, 10.61)	1.06 (0.72, 1.58)	1.07 (0.72, 1.58)	1.05 (0.71, 1.55)
UKB^f,g^					
Hearing impairment					
No	3,190 (219,599)	1.09 (1.05, 1.13)	Reference	Reference	Reference
Yes	3,734 (158,294)	1.79 (1.74, 1.85)	**1.17 (1.11, 1.22)**	**1.15 (1.10, 1.21)**	**1.12 (1.07, 1.17)**
Hearing aid use					
No	6,399 (366,898)	1.32 (1.28, 1.35)	Reference	Reference	Reference
Yes	525 (10,995)	3.74 (3.43, 4.07)	**1.31 (1.20, 1.44)**	**1.30 (1.19, 1.42)**	**1.23 (1.12, 1.34)**
Pooled data					
Hearing impairment^h^	159,740 (384,947)	NA	**1.16 (1.11, 1.22)**	**1.15 (1.10, 1.21)**	**1.12 (1.07, 1.17)**
Hearing aid use^i^	11,161 (384,947)	NA	**1.30 (1.19, 1.42)**	**1.29 (1.18, 1.40)**	**1.22 (1.12, 1.33)**

Values in bold are statistically significant. NA, not applicable.

^a^ Cause-specific Cox regression model adjusted for age (as timescale), sex, ethnicity, living alone (marital status in the WII cohort) and education.

^b^ Model 1 plus adjustment for BMI and health-related behaviors (smoking, alcohol consumption, fruit and vegetable consumption, and MET (min per week; moderate and vigorous physical activity in the WII study)).

^c^ Model 2 plus adjustment for the number of chronic conditions including coronary heart disease, stroke, hypertension, heart failure, diabetes, cancer, chronic kidney disease, chronic obstructive pulmonary disease, liver disease, depression, mental disorders, and arthritis or rheumatoid arthritis.

^d^ The median (IQR) follow-up is 24.8 (23.0–25.1) years.

^e^ The median (IQR) age at dementia diagnosis is 80.0 (75.6–84.2) years.

^f^ The median (IQR) follow-up is 13.6 (12.9–14.3) years.

^g^ The median (IQR) age at dementia diagnosis is 75.4 (72.2–78.3) years.

^h^HR is calculated using fixed-effects meta-analysis as no heterogeneity was found (Model 3: *I*^2^ = 0.00%; *Q* = 0.04; and *P* for *Q* statistic = 0.85).

^i^HR is calculated using fixed-effects meta-analysis as no heterogeneity was found (Model 3: *I*^2^ = 0.00%; *Q* = 0.60; and *P* for *Q* statistic = 0.44).

Analyses stratified by age at baseline (<55, ≥55 to <65 and ≥65 years) are shown in Supplementary Table 1. The prevalence of reported hearing impairment (18.6%, 22.0% and 23.8% in WII and 34.9%, 44.3% and 51.5% in UKB) and measured hearing impairment (6.2%, 11.8% and 20.5% in UKB) increased with age. There was no evidence of differences in associations between hearing impairment and incident dementia in these age groups.

Cross-classifying hearing impairment and hearing aid use (Supplementary Table 2) shows that in UKB among participants with hearing impairment, those using a hearing aid (HR 1.28; 95% CI 1.17, 1.41) or not (HR 1.10; 95% CI 1.04, 1.15) had higher dementia risk than those without hearing impairment or a hearing aid. Among those with hearing impairment, hearing aid users also had higher risk of dementia (HR 1.17; 95% CI 1.07 to 1.29). No associations were observed in WII.

UKB analyses stratified by the length of follow-up (≤10 years, >10 years) showed that associations of hearing impairment with incident dementia were not affected by the length of follow-up, but the association of hearing aid use with incident dementia was stronger when follow-up was ≤10 years (HR 1.32 versus 1.18) (Supplementary Table 3). The results for dementia subtypes in UKB showed similar associations for Alzheimer’s disease and vascular dementia (Supplementary Table 4).

Including early-onset (before 65 years) incident dementia cases in the analyses (Supplementary Tables 5 and 6) yielded similar results to that in the main analyses. Use of inverse propensity score weighting (IPSW) to address confounding modified the differences in baseline covariates between participants with and without a hearing aid (Supplementary Table 7), but the associations with incident dementia were similar with IPSW (Supplementary Tables 8 and 9).

In UKB, adjusting for hearing impairment severity (normal, insufficient and poor hearing) slightly attenuated the association of hearing aid use with incident dementia (Supplementary Table 10, Model 3). The interaction between hearing impairment or hearing aid with apolipoprotein E (ApoE) ε4 did not suggest differences in associations in ε4 carriers and noncarriers (all *P* for interaction >0.05); results of the stratified analyses are shown in Supplementary Table 11.

In UKB, 112,131 participants had both self-reported and objective (speech-in-noise (SiN)) measures of hearing impairment; there was a modest agreement between these two measures (all kappa coefficients <0.20) (Supplementary Table 12). Further analyses on participants with ‘concordant’ data on reported and measured hearing impairment (*n* = 67,586) show 11.19% to have both reported and objective hearing impairment and this group had higher risk of incident dementia (HR 1.27; 95% CI 1.10, 1.47) (Supplementary Table 13). Among hearing aid users, those with objective hearing impairment also had higher risk of dementia (HR 1.34; 95% CI 1.04, 1.74).

The PAF estimate, calculated only for potentially modifiable risk factors—here hearing impairment—was 2.79% in WII and 4.79% in UKB with a pooled estimate at 4.74% (Table 3). The PAF estimate in those with both reported and measured hearing impairment (concordant hearing impairment) in UKB was 2.94% and in those with objective hearing impairment it was 3.38%.

**Table 3:** PAFs for midlife hearing impairment

	Mean age (s.d.)	No. of men (%)	HR	Prevalence of exposure cases; total (%)	PAF (95% CI)^a^
Reported hearing impairment					
WII	55.9 (6.0)	4,973 (70.5)	1.14 (0.96, 1.35)	1,446; 7,054 (20.5)	2.79% (−0.82, 6.69)
UKB	56.2 (8.1)	179,650 (47.5)	**1.12 (1.07, 1.17)**	158,294; 377,893 (41.9)	4.79% (2.85, 6.65)
Pooled data^b^		184,623 (48.0)	**1.12 (1.07, 1.17)**	159,740; 384,947 (41.5)	4.74% (2.82, 6.59)
Concordant measures of hearing impairment (both reported and objective, UKB)					
Hearing impairment^c^	55.8 (8.3)	29,781 (44.1)	**1.27 (1.10, 1.47)**	7,560; 67,586 (11.2)	2.94% (1.11, 5.00)
Objective measure of hearing (UKB)					
SiN hearing impairment	56.5 (8.2)	54,852 (47.2)	**1.31 (1.17, 1.46)**	13,160; 116,256 (11.3)	3.38% (1.88, 5.10)

Bold formatting indicates statistical significance. HR values are calculated using cause-specific Cox regression.

^a^${\text{PAF}} = \frac{p ({\mathrm{RR}}-1)}{[1 + p ({\mathrm{RR}}-1)]}$ where *p* is the prevalence of the exposure and RR is the relative risk of dementia due to that exposure. Because the prevalence of dementia is <10%, HR was used as a proxy of RR.

^b^HR is calculated using fixed-effects meta-analysis as no heterogeneity was found (*I*^2^ = 0.00%; *Q* = 0.04; and *P* for *Q* statistic = 0.85).

^c^Both reported and SiN hearing impairment.

Taken together, our findings based on two large longitudinal studies show hearing impairment measured in ‘midlife’ to have a modest association with late-onset dementia. There is a growing body of research on potentially modifiable risk factors for dementia with a focus on midlife exposures, to reflect the decades-long preclinical phase of dementia^19^. Putative risk factors measured at older ages are affected by dementia-related physiological changes, making it difficult to draw ‘causal’ conclusions.

Recent meta-analyses have used PAF estimates, calculated from the prevalence of risk factors and the risk ratio, to highlight the ‘causal’ importance of hearing impairment for dementia. The risk ratios used in these meta-analyses were 1.35 (ref. ^5^) and 1.40 (refs. ^2,4^). The HR value for this association in our study using ‘midlife’ hearing impairment was much smaller; 1.14 in WII and 1.12 in UKB. Our estimates are similar to results from studies that measured hearing impairment in midlife (≤60 years): 1.08 (1.05, 1.12)^13^; 1.15 (1.06, 1.25)^20^; and 1.09 (0.97, 1.24)^21^. Few previous studies^11,12,21^ have a follow-up duration of more ≥20 years and only one of these had a measure of hearing impairment in participants <60 years where the risk ratio for dementia was 1.09 (0.97–1.24)^21^.

The prevalence estimates of hearing impairment used in the meta-analyses are quite high; 59% in one meta-analysis^2^, when the World Health Organization estimates for Europe are 10.9, 23.4, 42.0 and 56.5% in adults aged 60–69, 70–79, 80–89 and over 90 years, respectively^6^. A study that measured hearing impairment before age 60 reported much lower prevalence of 14.8% (ref. ^21^); in our study it was 18.9% and 37.2% in WII and UKB in participants <60 years at baseline. The prevalence of reported hearing impairment in the total UKB and WII populations was 41.9% and 20.5%; objectively measured hearing impairment in UKB was 11.3%. The PAF estimates in our study ranged from 2.79% to 4.79%; these are unweighted estimates because other potential risk factors have not been considered in PAF calculations. The unweighted estimates in recent meta-analyses for reported and measured hearing impairment are 15.6% (ref. ^3^) and 19.1% (ref. ^2^), the corresponding weighted PAF estimates being 7.2% and 7%.

Research on hearing impairment has led to interest in whether hearing aid wear mitigates the risk of dementia because of lower levels of social isolation, depression and cognitive load^22^. Some evidence from studies on older adults suggests a protective effect of hearing aid use^14–16,23^, but results from intervention studies are mostly null^17,18^. The age of participants, the reference group in the analyses and the length of follow-up may influence findings on hearing aid use. In UKB, there was some evidence of higher dementia risk in hearing aid users, as in a previous study using UKB data^24^, but stratifying by length of follow-up (Supplementary Table 3) yielded substantially smaller risk estimates for a longer follow-up (HR of 1.32 and 1.18 for a follow-up of ≤10 and >10 years, respectively). A single measure of hearing aid use, as in our analyses, may primarily be a marker of poorer hearing. In UKB, speech recognition in noise was worse in hearing aid users (mean −7.5 dB, s.d. 1.5) than in nonusers (−5.4 dB, s.d. 3.0). Adjustment for hearing impairment severity attenuated the association between hearing aid use and dementia in our analyses, supporting the hypothesis that hearing aid use is a marker of hearing impairment severity. Hearing aid use was not associated with the risk of dementia in WII where the follow-up was 25 years.

Age at measurement of hearing impairment (for prevalence), and the length of follow-up (to exclude reverse causation) play a role in estimates of PAF for late-onset dementia. The prevalence of hearing loss increases with age as a result of physiological changes and other external factors such as noise exposure, chronic conditions and lifestyle factors^22^. It is often progressive and may precede measurement of hearing impairment in studies based on older adults, not allowing judgments on whether it was already present in ‘midlife’. It has also been suggested that a common neurodegenerative mechanism, potentially resulting from genetic and vascular factors, as well as oxidative stress processes, may lead to both hearing impairment and dementia^25^. Dementia is a complex disease, with previous research showing changes in biomarkers leading to dementia commencing 15–20 years before clinical symptoms^19^. Trials on such a timescale are unfeasible but careful consideration of the age at assessment of risk factors and length of follow-up would allow better identification of risk factors for the prevention of dementia.

The main strength of this study is the use of two large cohorts with measures of ‘midlife’ hearing impairment, and a long follow-up, particularly in WII. We also adjusted for multiple confounders, including sociodemographic, behavioral and health factors. Limitations include self-reported hearing data in WII, although results using objective measures of hearing (SiN tests) in UKB were similar. However, both self-reported and objective hearing measures have limitations, including potential misclassification due to subjective perception or test conditions, which may bias the associations with dementia. Hearing aid use was only assessed at baseline, and changes over time were not examined. Hearing aid use may be inconsistent or ineffective and influenced by access, socioeconomic status or healthcare use, adding potential bias. Dementia ascertainment relied on electronic health records, offering data on dementia status for all participants but lacking biomarker confirmation. The UKB study and WII cohorts may be prone to selection bias, because the individuals recruited may represent those who have better health than the general population. Both cohort populations are largely composed of individuals with European, white British ancestry and a restricted age range, which may limit the generalizability of the findings. Confounding by indication may affect hearing aid results, and as with all observational studies, residual confounding may persist despite adjustments and the use of IPSW.

Dementia has a substantial impact on individuals, their families and healthcare systems, justifying ongoing therapeutic and prevention research. Risk factors measured during dementia’s long preclinical phase are unlikely to be ‘causal’, but whether modifying them delays the onset of dementia remains unclear. Our findings suggest a modest association between midlife hearing impairment and incident dementia, and do not support the unweighted PAF estimates of 15.6% (ref. ^3^) and 19.1% (ref. ^2^) in recent meta-analyses, in which the results on both the risk ratio and prevalence of hearing impairment are based primarily on data on older adults. Although our findings suggest weaker associations than those in recent meta-analyses, they do not refute a potential link between hearing impairment and dementia, highlighting the need for larger studies and consensus meta-analyses to refine risk estimates and PAF estimations of the association between ‘midlife’ hearing impairment and late-onset dementia.

## Methods

### Study population and design

The WII is an ongoing cohort study established in 1985 to 1988 among 10,308 men and women aged 35 to 55, working in 20 civil service departments in London^26^. Clinical examinations and self-administered questionnaires took place at baseline and subsequently every 4 or 5 years. Participants’ written consent and research ethics approval were renewed at each contact; the latest approval was from the Joint UCL/UCLH Committee on the Ethics of Human Research (reference number 85/0938). Of the 7,870 WII participants in the 1997–1999 wave, the baseline of our analysis, 803 participants with missing data on hearing measures or covariates, and 13 dementia cases diagnosed before age 65, were excluded, leading to a sample size of 7,054 participants.

The UKB study is a large population-based cohort study on 502,371 participants recruited between 2006 and 2010 among individuals aged 40–69 years registered with the UK National Health Service^27^. A wide range of information was collected at baseline via touchscreen questionnaires and clinical examinations. UKB obtained approval from the National Health Service North West Centre for Research Ethics Committee (reference number 11/NW/0382), and all participants gave written consent for participation. This research uses UK Biobank Resource under application number 96856. Of the 502,180 UKB participants, 123,398 with missing data on hearing measures or covariates were excluded. In addition, 228 prevalent cases of dementia at baseline and 661 cases with diagnosis before age 65, were also excluded. This led to a sample size of 377,893 UKB participants.

Participants in WII and UKB were not compensated for participating in this study. This study adheres to the Guidelines for Accurate and Transparent Health Estimate Reporting.

### Hearing impairment and hearing aid use

At the 1997–1999 wave of the WII study (age range 45–69 years), participants were asked to report: (1) whether they had difficulty hearing someone talking to them in a quiet room (with a hearing aid if normally worn); and (2) whether they had great difficulty following a conversation if there was background noise such as TV, radio or children (with a hearing aid if normally worn), with possible responses to both questions being ‘yes’ or ‘no’. Participants who responded ‘yes’ to either of the two questions were classified as having hearing impairment. Participants were also asked whether they wore a hearing aid and categorized as ‘yes’ or ‘no’.

In the UKB, data on hearing impairment status and hearing aid use were extracted from the baseline touchscreen questionnaire (2006 to 2010). Hearing impairment status was assessed by asking the participants: (1) whether they had any difficulty with their hearing and (2) whether they found it difficult to follow a conversation if there was background noise (such as TV, radio or children playing). Possible responses to both questions were ‘yes’, ‘no’ or ‘I am completely deaf’. Hearing impairment was defined as ‘yes’ or ‘I am completely deaf’ to either question. Participants were also asked whether they used a hearing aid most of the time, and hearing aid use was classified as ‘yes’ or ‘no’. This question was addressed to all participants except those who reported being deaf.

Objective measures of SiN hearing impairment in UKB (2006 to 2010) were ascertained using the digit triplet test for which participants were asked to remove their hearing aid, further details are available elsewhere^24,28^. This test determines the speech reception threshold in noise (SRTn), a measure of the ability to recognize speech in the presence of noise. The SRTn from the ear with better performance was used in the analyses unless data were available only for one ear. SRTn < −5.5 dB corresponds to normal hearing, SRTn ≥ −5.5 to SRTn ≤ −3.5 dB to insufficient hearing and SRTn > −3.5 dB to poor hearing^29^. SiN hearing impairment in our analyses was defined as SRTn ≥ −5.5 (including insufficient and poor hearing). 

### Dementia

All-cause dementia in both cohorts was ascertained by linkage to electronic health records databases using International Classification of Diseases 10th Revision codes F00-F03, F05.1, G30 and G31. Dementia cases were identified until 1 March 2023 in WII, and until 31 October, 31 August and 31 May 2022, in England, Scotland and Wales, respectively, in UKB. Ascertainment of all-cause dementia using the national Hospital Episode Statistics data has a sensitivity and specificity of 78.0% and 92.0% (ref. ^30^). However, sensitivity in our study is expected to be higher because of the inclusion of the mortality register (both studies), and the Mental Health Services Data Set (only WII study)^31^. The date of dementia was defined as the earliest date at which dementia was recorded in any register. Data on subtypes of dementia (Alzheimer’s disease and vascular dementia), available in UKB study, were used in additional analyses.

### Covariates

Sociodemographic measures in both cohorts included sex, education (low (less than high school)), intermediate (high school diploma) or high (university degree or professional qualification), ethnicity (white and minority ethnic groups) and marital status (married or cohabiting versus other in WII and living alone versus other in UKB). Covariates also included body mass index (BMI; calculated as the weight in kilograms divided by the square of the height in meters), health behaviors including alcohol consumption (no consumption, 1–14 units per week and >14 units per week), smoking (never, former or current smokers), fruit and vegetable consumption (less than daily, once a day, twice or more a day) and hours of moderate and vigorous physical activity per week in WII (metabolic equivalent of task (MET) in minutes per week in UKB), and the number of prevalent chronic diseases (including coronary heart disease, stroke, hypertension, heart failure, diabetes, cancer, chronic kidney disease, chronic obstructive pulmonary disease, liver disease, depression, mental disorders and arthritis or rheumatoid arthritis). ApoE genotype measure in UKB was based on two nucleotide polymorphisms (rs7412 and rs429358), and categorized into non-ApoE ε4 carriers and ApoE ε4 carriers.

### Statistics and reproducibility

No statistical methods were used to predetermine sample sizes in WII and UKB, but our sample sizes are similar to those reported in previous publications^24,26^. The proportional hazards assumption was verified by plotting Schoenfeld residuals, and then analyses were undertaken separately in each cohort using cause-specific Cox proportional hazards regression to take competing risk of death into account. Age was the timescale, and we first examined the association of hearing impairment and hearing aid use (separate models) with the incidence of all-cause dementia. Both hearing impairment and hearing aid use were then used to classify participants into three groups: (1) no hearing impairment and no hearing aid, (2) hearing impairment but no hearing aid and (3) hearing impairment and use of hearing aid. Because of the small numbers, participants who reported no hearing impairment but also reported using hearing aids were excluded from these analyses (*n* = 45 in WII and *n* = 64 in UKB). The association with incident dementia was examined using those with no hearing impairment and no hearing aid as the reference group. We then undertook analyses in only those participants who reported hearing impairment with the reference group being those not reporting use of hearing aid.

The start of follow-up for incident dementia in all analyses was the date of measurement of hearing (1997–1999 in WII and 2006–2010 in UKB). Prevalent dementia at baseline and early-onset cases (before age 65 years) were excluded from the main analyses (Extended Data Fig. 1). Participants were followed until the date of record of dementia, death or end of follow-up (1 March 2023 in WII, and 31 October 2022, 31 August 2022 and 31 May 2022 in England, Scotland and Wales, respectively, in UKB), whichever occurred first. The adjustment for covariates was in three steps, first sociodemographic variables (model 1), then also BMI and health-related behaviors (model 2) and then also the number of chronic diseases (model 3).

We calculated PAF, the fraction of all cases of dementia in a population attributable to hearing impairment in WII and UKB, with the formula ${\text{PAF}} = \frac{p ({\mathrm{RR}}-1)}{[1 + p ({\mathrm{RR}}-1)]}$ where *p* is the prevalence of the exposure and RR is the HR of dementia associated with the exposure.

Several additional analyses were undertaken to examine the robustness of the results, primarily in UKB owing to the small number of dementia cases in WII.

1. The association of hearing impairment and hearing aid use (separate models) was examined for dementia subtypes (Alzheimer’s disease and vascular dementia).
2. Age-stratified analyses (<55, ≥55 to <65 and ≥65 years at baseline) were undertaken to examine the role of age at the measure of hearing impairment in associations with dementia.
3. Early-onset dementia (onset <65 years) is genetically and phenotypically different from late-onset dementia leading us to remove these cases in the main analyses. We repeated the main analyses including early-onset dementia cases.
4. To examine the role of the length of follow-up, we repeated the analyses by splitting the follow-up as ≤10 and >10 years.
5. We repeated (both WII and UKB) the analyses on hearing aid use using IPSW to address confounding. This involved calculating a propensity score using a logistic regression model including all covariates to estimate the probability of being in the treatment group (hearing aid use). The inverse of this probability was then used as weights in the Cox regression.
6. To examine the role of hearing impairment severity in analyses on the association of hearing aid use with dementia, we further adjusted these analyses for hearing impairment severity (normal, insufficient and poor hearing) in a subsample of participants with concordant measures of reported and objective hearing impairment.
7. To investigate the role of ApoE ε4 in the association of hearing impairment and hearing aid use with incident dementia, analyses were stratified by ApoE ε4 carriage in a subsample of participants with data on ApoE.
8. We also examined the association of objective measures of hearing impairment with incident dementia.

All analyses were undertaken using Stata v.16.1 (StataCorp). A two-sided *P* value < 0.05 was considered statistically significant.

Data collection and analysis were not performed blind to the conditions of the experiments.

### Reporting summary

Further information on research design is available in the Nature Portfolio Reporting Summary linked to this article.

## Supplementary information

Supplementary Information Supplementary Tables 1 to 13.

Reporting Summary

Supplementary Code 1

## Data Availability

Whitehall II data are available through the Dementias Platform, UK, based at the University of Oxford. The details on how to access data are available at https://www.dementiasplatform.uk/. The UK Biobank data are available through a procedure described at https://www.ukbiobank.ac.uk/enable-your-research. Researchers registered with the UK Biobank can apply for access to the individual-level data by completing an application. For the research reported here, we applied for UK Biobank data using the UK Biobank Resource under Application Number 96856. This work uses data provided by patients and collected by the NHS as part of their care and support.

## Extended data

are available for this paper at 10.1038/s43587-025-00914-1.

## Code availability

All code used for this publication is available via GitHub at https://github.com/marcosmachado92/Hearing-impairment-and-Dementia.git.
