# Supplementary material for: Association of midlife hearing impairment and hearing aid use with incident dementia: analysis of two UK-based longitudinal cohort studies
Source: Nat Aging. 2025 Jul 1;5(9):1732–8. doi: 10.1038/s43587-025-00914-1 (PMC12443597; doi:10.1038/s43587-025-00914-1)
Supplement: Supplementary file 1 — Supplementary Information Supplementary Tables 1 to 13. [file 43587_2025_914_MOESM1_ESM.pdf]

nature aging

Supplementary information https://doi.org/10.1038/s43587-025-00914-1

Association of midlife hearing impairment and hearing aid use with incident dementia: analysis of two UK-based longitudinal cohort studies

In the format provided by the authors and unedited

Table S1. Association of hearing impairment with incident dementia in the Whitehall II and UK Biobank cohort studies, stratified by age at baseline.

	Prevalence of Hearing impairment	Dementia N dementia cases/Total	Dementia Rate/1000 person-years (95% CI)	Hazard ratio (95% CI)		
				Model 1[a]	Model 2[b]	Model 3[c]
**WHITEHALL II (reported hearing impairment)**						
**<55 years**						
No hearing impairment	18.6%	84/2,790	1.26 (1.01, 1.56)	Ref.	Ref.	Ref.
Hearing impairment		21/638	1.40 (0.91, 2.15)	1.09 (0.67, 1.76)	1.12 (0.69, 1.81)	1.11 (0.68, 1.79)
**≥55 to <65 years**						
No hearing impairment	22.0%	338/2,373	6.53 (5.87, 7.26)	Ref.	Ref.	Ref.
Hearing impairment		115/669	8.00 (6.67, 9.61)	1.15 (0.93, 1.42)	1.13 (0.91, 1.40)	1.12 (0.90, 1.38)
**≥65 years**						
No hearing impairment	23.8%	97/445	11.54 (9.46, 14.09)	Ref.	Ref.	Ref.
Hearing impairment		37/139	14.17 (10.27, 19.56)	1.19 (0.81, 1.74)	1.19 (0.80, 1.76)	1.19 (0.80, 1.76)
**UK BIOBANK (reported hearing impairment)**						
**<55 years**						
No hearing impairment	34.9%	39/97,943	0.03 (0.02, 0.04)	Ref.	Ref.	Ref.
Hearing impairment		27/52,540	0.04 (0.03, 0.05)	0.91 (0.56, 1.50)	0.91 (0.55, 1.49)	0.86 (0.53, 1.42)
**≥55 to <65 years**						
No hearing impairment	44.3%	1,285/88,296	1.10 (1.04, 1.16)	Ref.	Ref.	Ref.
Hearing impairment		1,356/70,283	1.46 (1.39, 1.54)	**1.22 (1.13, 1.31)**	**1.20 (1.11, 1.29)**	**1.15 (1.07, 1.25)**
**≥65 years**						
No hearing impairment	51.5%	1,866/33,360	4.40 (4.20, 4.60)	Ref.	Ref.	Ref.
Hearing impairment		2,351/35,471	5.28 (5.07, 5.50)	**1.15 (1.08, 1.23)**	**1.14 (1.07, 1.22)**	**1.11 (1.05, 1.18)**
**UK BIOBANK (SiN measured hearing impairment)**						
**<55 years**						
No hearing impairment	6.2%	11/42,140	0.02 (0.01, 0.04)	Ref.	Ref.	Ref.
Hearing impairment		0/2,768	-	-	-	-
**≥55 to <65 years**						
No hearing impairment	11.8%	531/43,032	0.99 (0.91, 1.08)	Ref.	Ref.	Ref.
Hearing impairment		132/5,773	1.87 (1.58, 2.22)	**1.55 (1.28, 1.88)**	**1.48 (1.22, 1.80)**	**1.40 (1.15, 1.70)**
**≥65 years**						
No hearing impairment	20.5%	795/17,924	3.69 (3.44, 3.95)	Ref.	Ref.	Ref.
Hearing impairment		284/4,619	5.23 (4.65, 5.87)	**1.35 (1.18, 1.55)**	**1.34 (1.17, 1.53)**	**1.31 (1.14, 1.50)**

SiN: speech-in-noise. Bold formatting indicates statistical significance.

[a] Cause-specific Cox regression model adjusted for age (as time-scale), sex, ethnicity, living alone (marital status in the Whitehall II cohort study), and education.

[b] Model 1 plus adjustment for body mass index and health-related behaviors (smoking, alcohol consumption, fruit and vegetables consumption, and MET min/week (moderate and vigorous physical activity in the Whitehall II study)).

[c] Model 2 plus adjustment for the number of chronic conditions including coronary heart disease, stroke, hypertension, heart failure, diabetes, cancer, chronic kidney disease, chronic obstructive pulmonary disease, liver disease, depression, mental disorders, and arthritis/rheumatoid arthritis.

Table S2. Associations of hearing aid use, in those with hearing impairment, with incident dementia in Whitehall II and the UK Biobank cohort studies.

	N dementia cases/Total	Hazard ratio (95% CI)		
		Model 1[a]	Model 2[b]	Model 3[c]
**WHITEHALL II[d]**				
No hearing impairment, no hearing aid	512/5,563	Ref.	Ref.	Ref.
Hearing impairment, no hearing aid	154/1,325	1.16 (0.96, 1.38)	1.15 (0.96, 1.38)	1.14 (0.95, 1.37)
Hearing impairment, hearing aid	19/121	1.11 (0.70, 1.76)	1.11 (0.70, 1.76)	1.10 (0.69, 1.74)
**UK BIOBANK[e]**				
No hearing impairment, no hearing aid	3,188/219,535	Ref.	Ref.	Ref.
Hearing impairment, no hearing aid	3,211/147,363	**1.14 (1.08, 1.19)**	**1.13 (1.07, 1.18)**	**1.10 (1.04, 1.15)**
Hearing impairment, hearing aid	523/10,931	**1.40 (1.28, 1.54)**	**1.38 (1.26, 1.51)**	**1.28 (1.17, 1.41)**
**ANALYSES ON HEARING AID USE RESTRICTED TO PARTICIPANTS REPORTING HEARING IMPAIRMENT**				
**WHITEHALL II[f]**				
No hearing aid	154/1,325	Ref.	Ref.	Ref.
Hearing aid user	19/121	0.95 (0.59, 1.53)	0.97 (0.60, 1.57)	0.97 (0.60, 1.57)
**UK BIOBANK[g]**				
No hearing aid	3,211/147,363	Ref.	Ref.	Ref.
Hearing aid user	523/10,931	**1.24 (1.13, 1.36)**	**1.23 (1.12, 1.35)**	**1.17 (1.07, 1.29)**

Bold formatting indicates statistical significance.

[a] Cause-specific Cox regression model adjusted for age (as time-scale), sex, ethnicity, living alone (marital status in the Whitehall II cohort), and education.

[b] Model 1 plus adjustment for body mass index and health-related behaviors (smoking, alcohol consumption, fruit and vegetables consumption, and MET min/week (moderate and vigorous physical activity in the Whitehall II study)).

[c] Model 2 plus adjustment for the number of chronic conditions including coronary heart disease, stroke, hypertension, heart failure, diabetes, cancer, chronic kidney disease, chronic obstructive pulmonary disease, liver disease, depression, mental disorders, and arthritis/rheumatoid arthritis.

[d] The median (IQR) follow-up is 24.8 (23.1-25.1) years.

[e] The median (IQR) follow-up is 13.6 (12.9-14.3) years.

[f] The median (IQR) follow-up is 24.8 (23.1-25.1) years.

[g] The median (IQR) follow-up is 13.6 (12.8-14.3) years.

Table S3. Association of hearing impairment and hearing aid use with incident dementia in UK Biobank, stratified by the length of follow-up.

	Follow-up ≤10 years			Follow-up >10 years		
	N dementia cases/Total	Rate/1000 person-years (95% CI)	Hazard ratio (95% CI)[a]	N dementia cases/Total	Rate/1000 person-years (95% CI)	Hazard ratio (95% CI)[a]
**Hearing impairment**						
No	1,244/219,599	0.58 (0.55, 0.61)	Ref.	1,946/209,479	2.54 (2.43, 2.66)	Ref.
Yes	1,537/158,294	1.00 (0.95, 1.05)	**1.12 (1.04, 1.21)**	2,197/148,521	4.07 (3.91, 4.25)	**1.13 (1.06, 1.20)**
**Hearing aid use**						
No	2,538/366,898	0.71 (0.68, 0.73)	Ref.	3,861/348,114	3.04 (2.95, 3.14)	Ref.
Yes	243/10,995	2.31 (2.03, 2.62)	**1.32 (1.16, 1.51)**	282/9,886	8.03 (7.14, 9.02)	**1.18 (1.05, 1.34)**
**Hearing aid use in those with hearing impairment**						
No hearing impairment, no hearing aid	1,243/219,535	0.58 (0.55, 0.61)	Ref.	1,945/209,418	2.54 (2.43, 2.66)	Ref.
Hearing impairment, no hearing aid	1,295/147,363	0.90 (0.85, 0.95)	1.09 (1.00, 1.18)	1,916/138,696	3.80 (3.63, 3.97)	**1.11 (1.04, 1.18)**
Hearing impairment, hearing aid	242/10,931	2.31 (2.04, 2.62)	**1.38 (1.20, 1.59)**	281/9,825	8.04 (7.15, 9.03)	**1.25 (1.10, 1.42)**
**ANALYSES RESTRICTED TO PARTICIPANTS REPORTING HEARING IMPAIRMENT**						
No hearing aid	1,295/147,363	0.90 (0.85, 0.95)	Ref.	1,916/138,696	3.80 (3.63, 3.97)	Ref.
Hearing aid use	242/10,931	2.31 (2.04, 2.62)	**1.27 (1.11, 1.46)**	281/9,825	8.04 (7.15, 9.03)	1.13 (0.99, 1.28)

Bold formatting indicates statistical significance.

[a] Cause-specific Cox regression model adjusted for age (as time-scale), sex, ethnicity, living alone, education, body mass index, health-related behaviors (smoking, alcohol consumption, fruit and vegetables consumption, and MET min/week), and number of chronic conditions including coronary heart disease, stroke, hypertension, heart failure, diabetes, cancer, chronic kidney disease, chronic obstructive pulmonary disease, liver disease, depression, mental disorders, and arthritis/rheumatoid arthritis.

Table S4. Association of hearing impairment and hearing aid use with incident Alzheimer's disease and vascular dementia in the UK Biobank cohort study.

	N dementia cases/Total	Rate/1000 person-years (95% CI)	Hazard ratio (95% CI)[a]		
			Model 1[b]	Model 2[c]	Model 3[d]
**Alzheimer's disease[e]**					
Hearing impairment					
No	1,326/219,599	0.45 (0.43, 0.48)	Ref.	Ref.	Ref.
Yes	1,488/158,294	0.71 (0.68, 0.75)	**1.15 (1.07, 1.24)**	**1.14 (1.06, 1.23)**	**1.12 (1.04, 1.20)**
Hearing aid use					
No	2,610/366,898	0.53 (0.52, 0.56)	Ref.	Ref.	Ref.
Yes	204/10,995	1.45 (1.27, 1.67)	**1.26 (1.09, 1.46)**	**1.25 (1.09, 1.45)**	**1.20 (1.04, 1.39)**
**Vascular dementia[f]**					
Hearing impairment					
No	586/219,599	0.20 (0.19, 0.22)	Ref.	Ref.	Ref.
Yes	782/158,294	0.38 (0.35, 0.40)	**1.28 (1.15, 1.43)**	**1.26 (1.13, 1.40)**	**1.19 (1.07, 1.33)**
Hearing aid use					
No	1,243/366,898	0.26 (0.24, 0.27)	Ref.	Ref.	Ref.
Yes	125/10,995	0.89 (0.75, 1.06)	**1.55 (1.29, 1.86)**	**1.51 (1.26, 1.82)**	**1.37 (1.14, 1.65)**

Bold formatting indicates statistical significance.

[a] The median (IQR) follow-up is 13.6 (12.9-14.3).

[b] Cause-specific Cox regression model adjusted for age (as time-scale), sex, ethnicity, living alone, and education.

[c] Model 1 plus adjustment for body mass index and health-related behaviors (smoking, alcohol consumption, fruit and vegetables consumption, and MET min/week).

[d] Model 2 plus adjustment for the number of chronic conditions including coronary heart disease, stroke, hypertension, heart failure, diabetes, cancer, chronic kidney disease, chronic obstructive pulmonary disease, liver disease, depression, mental disorders, and arthritis/rheumatoid arthritis.

[e] The median (IQR) age at Alzheimer's disease diagnosis is 75.6 (72.4-78.5).

[f] The median (IQR) age at vascular dementia diagnosis is 75.5 (72.0-78.1).

Table S5. Associations of reported hearing impairment and hearing aid use with incident dementia in the Whitehall II and UK Biobank cohort studies including incident dementia cases occurring before 65 years.

	N dementia cases/Total	Rate/1000 person-years (95% CI)	Hazard ratio (95% CI)		
			**Model 1[a]**	**Model 2[b]**	**Model 3[c]**
**WHITEHALL II[d, e]**					
**Hearing impairment**					
No	528/5,617	4.16 (3.82, 4.53)	Ref.	Ref.	Ref.
Yes	177/1,450	5.53 (4.77, 6.40)	1.16 (0.98, 1.38)	1.16 (0.97, 1.37)	1.15 (0.97, 1.36)
**Hearing aid use**					
No	679/6,901	4.37 (4.05, 4.71)	Ref.	Ref.	Ref.
Yes	26/166	7.22 (4.92, 10.61)	1.05 (0.71, 1.56)	1.06 (0.71, 1.57)	1.04 (0.70, 1.53)
**UK BIOBANK[f, g]**					
**Hearing impairment**					
No	3,512/219,921	1.20 (1.16, 1.24)	Ref.	Ref.	Ref.
Yes	4,073/158,633	1.95 (1.89, 2.01)	**1.19 (1.14, 1.25)**	**1.18 (1.13, 1.23)**	**1.14 (1.09, 1.19)**
**Hearing aid use**					
No	7,030/367,529	1.44 (1.41, 1.48)	Ref.	Ref.	Ref.
Yes	555/11,025	3.94 (3.63, 4.29)	**1.35 (1.24, 1.47)**	**1.33 (1.22, 1.45)**	**1.25 (1.14, 1.36)**

Bold formatting indicates statistical significance.

[a] Cause-specific Cox regression model adjusted for age (as time-scale), sex, ethnicity, living alone (marital status in the Whitehall II cohort), and education.

[b] Model 1 plus adjustment for body mass index and health-related behaviors (smoking, alcohol consumption, fruit and vegetables consumption, and MET min/week (moderate and vigorous physical activity in the Whitehall II study)).

[c] Model 2 plus adjustment for the number of chronic conditions including coronary heart disease, stroke, hypertension, heart failure, diabetes, cancer, chronic kidney disease, chronic obstructive pulmonary disease, liver disease, depression, mental disorders, and arthritis/rheumatoid arthritis.

[d] The median (IQR) follow-up is 24.8 (23.0-25.1) years. [e] The median (IQR) age at dementia diagnosis is 79.9 (75.3-84.0) years.

[f] The median (IQR) follow-up is 13.6 (12.9-14.3) years. [g] The median (IQR) age at dementia diagnosis is 74.9 (70.9-78.0) years.

Table S6. Association of hearing aid use, in those with hearing impairment, with incident dementia in the Whitehall II and UK Biobank cohort studies including incident dementia cases occurring before 65 years.

	N dementia cases/Total	Hazard ratio (95% CI)		
		**Model 1[a]**	**Model 2[b]**	**Model 3[c]**
**WHITEHALL II[d]**				
No hearing impairment, no hearing aid	521/5,572	Ref.	Ref.	Ref.
Hearing impairment, no hearing aid	158/1,329	1.17 (0.98, 1.40)	1.16 (0.97, 1.39)	1.15 (0.96, 1.38)
Hearing impairment, hearing aid	19/121	1.10 (0.70, 1.75)	1.10 (0.70, 1.75)	1.09 (0.69, 1.72)
**UK BIOBANK[e]**				
No hearing impairment, no hearing aid	3,510/219,857	Ref.	Ref.	Ref.
Hearing impairment, no hearing aid	3,520/147,672	**1.16 (1.11, 1.22)**	**1.15 (1.10, 1.20)**	**1.11 (1.06, 1.17)**
Hearing impairment, hearing aid	553/10,961	**1.45 (1.33, 1.59)**	**1.43 (1.30, 1.56)**	**1.32 (1.20, 1.44)**
**ANALYSES ON HEARING AID USE RESTRICTED TO PARTICIPANTS REPORTING HEARING IMPAIRMENT**				
**WHITEHALL II[f]**				
No hearing aid	158/1,329	Ref.	Ref.	Ref.
Hearing aid user	19/121	0.93 (0.58, 1.51)	0.96 (0.59, 1.55)	0.95 (0.59, 1.54)
**UK BIOBANK[g]**				
No hearing aid	3,520/147,672	Ref.	Ref.	Ref.
Hearing aid user	553/10,961	**1.26 (1.15, 1.38)**	**1.25 (1.15, 1.37)**	**1.19 (1.09, 1.31)**

Bold formatting indicates statistical significance.

[a] Cause-specific Cox regression model adjusted for age (as time-scale), sex, ethnicity, living alone (marital status in the Whitehall II cohort study), and education.

[b] Model 1 plus adjustment for body mass index and health-related behaviors (smoking, alcohol consumption, fruit and vegetables consumption, and MET min/week (moderate and vigorous physical activity in the Whitehall II study)).

[c] Model 2 plus adjustment for the number of chronic conditions including coronary heart disease, stroke, hypertension, heart failure, diabetes, cancer, chronic kidney disease, chronic obstructive pulmonary disease, liver disease, depression, mental disorders, and arthritis/rheumatoid arthritis.

[d] The median (IQR) follow-up is 24.8 (23.0-25.1) years. [e] The median (IQR) follow-up is 13.6 (12.9-14.3) years. [f] The median (IQR) follow-up is 24.8 (21.4-25.1) years. [g] The median (IQR) follow-up is 13.6 (12.8-14.3) years.

Table S7. Standardized differences between hearing aid users and non-users as a function of baseline covariates, before and after use of inverse propensity score weighting (IPSW) in Whitehall II and UK Biobank cohort studies.

	WHITEHALL II		UK BIOBANK	
	**Standardized difference before IPSW**	**Standardized difference after IPSW**	**Standardized difference before IPSW**	**Standardized difference after IPSW**
**Age**	0.66	-0.01	-0.79	0.01
**Sex**	-0.03	-0.14	-0.19	0.07
**Ethnicity**	0.08	-0.06	0.10	-0.01
**Marital status (living alone)**	-0.05	-0.04	-0.01	-0.03
**Education**				
Low	0.21	0.04	0.31	0.01
Intermediate	-0.07	0.01	-0.04	0.01
High	-0.17	-0.06	-0.21	-0.02
**BMI**				
Underweight	-0.11	-0.04	-0.15	-0.01
Normal weight	0.05	-0.05	-0.02	0.01
Overweight	-0.01	-0.07	0.04	-0.02
Obesity	0.15	-0.03	0.11	0.03
**Alcohol consumption**				
No consumption	-0.07	-0.01	0.09	0.06
1-14 units/wk	0.10	0.09	0.01	-0.01
>14 units/wk	-0.05	-0.08	-0.10	-0.05
**Smoking**				
Never smoker	-0.11	0.07	-0.16	0.03
Current smoker	0.10	-0.07	0.19	-0.03
Former smoker	0.01	-0.01	-0.04	0.01
**Fruit and vegetables consumption**				
Less than daily	-0.14	0.04	-0.02	-0.01
Once a day	-0.05	-0.02	-0.02	0.01
Twice or more a day	0.17	-0.02	0.03	-0.01
**Physical activity**	0.14	-0.09	-0.01	-0.01
**Number of chronic diseases**	0.24	-0.06	0.44	0.02

IPSW: inverse propensity score weighting

Table S8. Association of reported hearing aid use with incident dementia in the Whitehall II and UK Biobank cohort studies using inverse propensity score weighting.

	N dementia cases/Total	Rate/1000 person-years (95% CI)	Hazard ratio (95% CI)		
			Model 1[a]	Model 2[b]	Model 3[c]
**WHITEHALL II[d, e]**					
No	666/6,888	4.34 (4.04, 4.68)	Ref.	Ref.	Ref.
Yes	26/166	5.62 (3.44, 9.74)	1.29 (0.76, 2.16)	1.27 (0.75, 2.13)	1.27 (0.76, 2.14)
**UK BIOBANK[f, g]**					
No	6,399/366,898	1.36 (1.33, 1.39)	Ref.	Ref.	Ref.
Yes	525/10,995	1.77 (1.61, 1.95)	**1.26 (1.14, 1.39)**	**1.27 (1.15, 1.40)**	**1.28 (1.16, 1.41)**

Bold formatting indicates statistical significance.

[a] Cause-specific Cox regression model adjusted for age (as time-scale), sex, ethnicity, living alone (marital status in the Whitehall II cohort study), and education.

[b] Model 1 plus adjustment for body mass index and health-related behaviors (smoking, alcohol consumption, fruit and vegetables consumption, and MET min/week (moderate and vigorous physical activity in the Whitehall II study)).

[c] Model 2 plus adjustment for the number of chronic conditions including coronary heart disease, stroke, hypertension, heart failure, diabetes, cancer, chronic kidney disease, chronic obstructive pulmonary disease, liver disease, depression, mental disorders, and arthritis/rheumatoid arthritis.

[d] The median (IQR) follow-up is 24.8 (23.0-25.1) years. [e] The median (IQR) age at dementia diagnosis is 80.0 (75.6-84.2) years. [f] The median (IQR) follow-up is 13.6 (12.9-14.3) years. [g] The median (IQR) age at dementia diagnosis is 75.4 (72.2-78.3) years.

Table S9. Association of hearing aid use and hearing impairment categories with incident dementia in the Whitehall II and UK Biobank cohort studies using inverse propensity score weighting.

	N dementia cases/Total	Hazard ratio (95% CI)		
		Model 1[a]	Model 2[b]	Model 3[c]
**WHITEHALL II**[d]				
No hearing impairment, no hearing aid	512/5,563	Ref.	Ref.	Ref.
Hearing impairment, no hearing aid	154/1,325	1.15 (0.96, 1.39)	1.15 (0.96, 1.38)	1.14 (0.95, 1.37)
Hearing impairment, hearing aid	19/121	1.38 (0.74, 2.57)	1.37 (0.74, 2.54)	1.38 (0.74, 2.56)
**UK BIOBANK**[e]				
No hearing impairment, no hearing aid	3,188/219,535	Ref.	Ref.	Ref.
Hearing impairment, no hearing aid	3,211/147,363	**1.13 (1.08, 1.19)**	**1.12 (1.07, 1.18)**	**1.09 (1.04, 1.15)**
Hearing impairment, hearing aid	523/10,931	**1.34 (1.21, 1.48)**	**1.34 (1.22, 1.49)**	**1.34 (1.21, 1.48)**
**ANALYSES ON HEARING AID USE RESTRICTED TO PARTICIPANTS REPORTING HEARING IMPAIRMENT**				
**WHITEHALL II**[f]				
No hearing aid	154/1,325	Ref.	Ref.	Ref.
Hearing aid user	19/121	1.14 (0.60, 2.13)	1.13 (0.60, 2.14)	1.15 (0.61, 2.17)
**UK BIOBANK**[g]				
No hearing aid	3,211/147,363	Ref.	Ref.	Ref.
Hearing aid user	523/10,931	**1.18 (1.07, 1.30)**	**1.19 (1.08, 1.32)**	**1.22 (1.10, 1.35)**

Bold formatting indicates statistical significance.

[a] Cause-specific Cox regression model adjusted for age (as time-scale), sex, ethnicity, living alone (marital status in the Whitehall II study), and education.

[b] Model 1 plus adjustment for body mass index and health-related behaviors (smoking, alcohol consumption, fruit and vegetables consumption, and MET min/week (moderate and vigorous physical activity in the Whitehall II study)).

[c] Model 2 plus adjustment for the number of chronic conditions including coronary heart disease, stroke, hypertension, heart failure, diabetes, cancer, chronic kidney disease, chronic obstructive pulmonary disease, liver disease, depression, mental disorders, and arthritis/rheumatoid arthritis.

[d] The median (IQR) follow-up is 24.8 (23.1-25.1) years.[e] The median (IQR) follow-up is 13.6 (12.9-14.3) years. [f] The median (IQR) follow-up is 24.8 (23.1-25.1) years. [g] The median (IQR) follow-up is 13.6 (12.8-14.3) years.

Table S10. Association of "concordant" hearing aid use with incident dementia in the UK Biobank cohort study adjusted for objectively measured hearing impairment severity.

	N dementia cases/Total	Rate/1000 person-years (95% CI)	Hazard ratio (95% CI)		
			Model 1[a]	Model 2[b]	Model 3[c]
**Hearing aid use**					
No hearing aid and no SiN hearing impairment	1,237/97,633	1.02 (0.96, 1.08)	Ref.	Ref.	Ref.
Hearing aid use and SiN hearing impairment	62/1,373	3.82 (2.98, 4.90)	**1.45 (1.12, 1.87)**	**1.34 (1.04, 1.74)**	1.40 (0.96, 2.04)

SiN: speech-in-noise. Bold formatting indicates statistical significance.

[a] Cause-specific Cox regression model adjusted for age (as time-scale), sex, ethnicity, living alone, and education.

[b] Model 1 plus adjustment for body mass index, health-related behaviors (smoking, alcohol consumption, fruit and vegetables consumption, and MET min/week), and number of chronic conditions including coronary heart disease, stroke, hypertension, heart failure, diabetes, cancer, chronic kidney disease, chronic obstructive pulmonary disease, liver disease, depression, mental disorders, and arthritis/rheumatoid arthritis.

[c] Model 2 plus adjustment for hearing impairment severity (normal, insufficient, and poor hearing).

Table S11. Association of hearing impairment and hearing aid use with incident dementia in the UK Biobank cohort study, stratified by ApoE e4 status.

	N dementia cases/Total	Rate/1000 person-years (95% CI)	Hazard ratio (95% CI)[d]		
			Model 1[a]	Model 2[b]	Model 3[c]
**Hearing impairment**					
Non-ApoE e4 carriers					
No	1,232/130,083	0.71 (0.67, 0.75)	Ref.	Ref.	Ref.
Yes	1,459/93,625	1.18 (1.12, 1.24)	**1.15 (1.07, 1.25)**	**1.14 (1.05, 1.23)**	**1.10 (1.02, 1.18)**
ApoE e4 carriers					
No	1,368/52,030	1.98 (1.88, 2.09)	Ref.	Ref.	Ref.
Yes	1,576/37,289	3.22 (3.06, 3.38)	**1.17 (1.09, 1.26)**	**1.16 (1.08, 1.25)**	**1.13 (1.05, 1.22)**
**Hearing aid use**					
Non-ApoE e4 carriers					
No	2,467/217,185	0.85 (0.82, 0.89)	Ref.	Ref.	Ref.
Yes	224/6,523	2.67 (2.34, 3.04)	**1.44 (1.26, 1.66)**	**1.42 (1.24, 1.63)**	**1.32 (1.15, 1.51)**
ApoE e4 carriers					
No	2,731/86,722	2.38 (2.29, 2.47)	Ref.	Ref.	Ref.
Yes	213/2,597	6.46 (5.65, 7.38)	**1.22 (1.06, 1.40)**	**1.21 (1.05, 1.39)**	1.15 (1.00, 1.32)

Bold formatting indicates statistical significance.

[a] Cause-specific Cox regression model adjusted for age (as time-scale), sex, ethnicity, living alone, and education.

[b] Model 1 plus adjustment for body mass index and health-related behaviors (smoking, alcohol consumption, fruit and vegetables consumption, and MET min/week).

[c] Model 2 plus adjustment for the number of chronic conditions including coronary heart disease, stroke, hypertension, heart failure, diabetes, cancer, chronic kidney disease, chronic obstructive pulmonary disease, liver disease, depression, mental disorders, and arthritis/rheumatoid arthritis.

[d] The median (IQR) follow-up is 13.6 (12.9-14.3) in ApoE e4 non-carriers and 13.6 (12.9-14.3) in ApoE e4 carriers.

Table S12. Concordance between reported (hearing impairment and hearing aid use) and objective measures of speech-in-noise hearing impairment in the UK Biobank cohort study.

	Speech-in-noise hearing impairment[a]		Kappa statistic
	**No**	**Yes**	
**Hearing impairment**			
No	60,026	5,205	0.09
Yes	39,340	7,560	
**Hearing aid use**			
No	97,633	11,392	0.13
Yes	1,733	1,373	

[a] Speech-in-noise hearing impairment defined as insufficient or poor hearing in the speech-in-noise test.

Table S13. Association of reported hearing impairment and hearing aid use ("concordant" with measured hearing impairment) with incident dementia in the UK Biobank cohort study.

	N dementia cases/Total	Rate/1000 person-years (95% CI)	Hazard ratio (95% CI)	
			Model 1[a]	Model 2[b]
**Hearing impairment**				
No reported or speech-in-noise (SiN) hearing impairment	671/60,026	0.90 (0.83, 0.97)	Ref.	Ref.
Both reported and SiN hearing impairment	273/7,560	2.99 (2.66, 3.37)	**1.39 (1.20, 1.61)**	**1.27 (1.10, 1.47)**
**Hearing aid use**				
No hearing aid and no SiN hearing impairment	1,237/97,633	1.02 (0.96, 1.08)	Ref.	Ref.
Hearing aid use and SiN hearing impairment	62/1,373	3.82 (2.98, 4.90)	**1.45 (1.12, 1.87)**	**1.34 (1.04, 1.74)**

SiN: speech-in-noise. Bold formatting indicates statistical significance.

[a] Cause-specific Cox regression model adjusted for age (as time-scale), sex, ethnicity, living alone, and education.

[b] Model 1 plus adjustment for body mass index, health-related behaviors (smoking, alcohol consumption, fruit and vegetables consumption, and MET min/week), and number of chronic conditions including coronary heart disease, stroke, hypertension, heart failure, diabetes, cancer, chronic kidney disease, chronic obstructive pulmonary disease, liver disease, depression, mental disorders, and arthritis/rheumatoid arthritis.
