# Supplementary material for: Association of midlife hearing impairment and hearing aid use with incident dementia: analysis of two UK-based longitudinal cohort studies
Source: Nat Aging. 2025 Jul 1;5(9):1732–8. doi: 10.1038/s43587-025-00914-1 (PMC12443597; doi:10.1038/s43587-025-00914-1)
Supplement: Supplementary file 2 — Reporting Summary [file 43587_2025_914_MOESM2_ESM.pdf]

nature portfolio

Corresponding author(s): Severine Sabia

Last updated by author(s): May 8, 2025

Reporting Summary

Nature Portfolio wishes to improve the reproducibility of the work that we publish. This form provides structure for consistency and transparency in reporting. For further information on Nature Portfolio policies, see our Editorial Policies and the Editorial Policy Checklist.

Statistics

For all statistical analyses, confirm that the following items are present in the figure legend, table legend, main text, or Methods section.

n/a	Confirmed	
☐	☒	The exact sample size (*n*) for each experimental group/condition, given as a discrete number and unit of measurement
☐	☒	A statement on whether measurements were taken from distinct samples or whether the same sample was measured repeatedly
☐	☒	The statistical test(s) used AND whether they are one- or two-sided<br>*Only common tests should be described solely by name; describe more complex techniques in the Methods section.*
☐	☒	A description of all covariates tested
☐	☒	A description of any assumptions or corrections, such as tests of normality and adjustment for multiple comparisons
☐	☒	A full description of the statistical parameters including central tendency (e.g. means) or other basic estimates (e.g. regression coefficient) AND variation (e.g. standard deviation) or associated estimates of uncertainty (e.g. confidence intervals)
☐	☒	For null hypothesis testing, the test statistic (e.g. *F*, *t*, *r*) with confidence intervals, effect sizes, degrees of freedom and *P* value noted<br>*Give P values as exact values whenever suitable.*
☒	☐	For Bayesian analysis, information on the choice of priors and Markov chain Monte Carlo settings
☒	☐	For hierarchical and complex designs, identification of the appropriate level for tests and full reporting of outcomes
☐	☒	Estimates of effect sizes (e.g. Cohen's *d*, Pearson's *r*), indicating how they were calculated

Our web collection on statistics for biologists contains articles on many of the points above.

Software and code

Policy information about availability of computer code

Data collection	These are secondary data analyses based on data from two cohort studies.
Data analysis	Stata 16.1. for all analyses.

For manuscripts utilizing custom algorithms or software that are central to the research but not yet described in published literature, software must be made available to editors and reviewers. We strongly encourage code deposition in a community repository (e.g. GitHub). See the Nature Portfolio guidelines for submitting code & software for further information.

Data

Policy information about availability of data

All manuscripts must include a data availability statement. This statement should provide the following information, where applicable:

- Accession codes, unique identifiers, or web links for publicly available datasets
- A description of any restrictions on data availability
- For clinical datasets or third party data, please ensure that the statement adheres to our policy

Whitehall II and UK Biobank data cannot be made available on the journal's website publicly because of constraints dictated by the study's ethics approval and IRB restrictions.
Whitehall II data are are available through the Dementias Platform, UK based at University of Oxford. The details on how to access data are available at https://www.dementiasplatform.uk/

UK Biobank data are available through a procedure described here, https://www.ukbiobank.ac.uk/enable-your-research
For the research reported here we applied for UK Biobank data using the UK Biobank Resource under Application Number 96856. This work uses data provided by patients and collected by the NHS as part of their care and support.

Research involving human participants, their data, or biological material

Policy information about studies with human participants or human data. See also policy information about sex, gender (identity/presentation), and sexual orientation and race, ethnicity and racism.

Reporting on sex and gender	We tested for the interaction between the exposure (hearing impairment and hearing aid use) and sex, and the lack of interaction led us to combine men and women in the analyses. Analyses were adjusted for sex.
Reporting on race, ethnicity, or other socially relevant groupings	We tested for the interaction between the exposure (hearing impairment and hearing aid use) and ethnicity, and the lack of interaction led us to combine all participants in the analyses. Analyses were adjusted for ethnicity.
Population characteristics	Analyses in WII were based on 7,054 participants (mean age ± SD 55.9 ± 6.0 years; 29.5% women); 692 cases of incident dementia were recorded during a median (IQR) follow-up of 24.8 (23.0 to 25.1) years. In UKB, 377,893 participants (mean age ± SD 56.3 ± 8.1 years; 52.5% women) were included in the analyses and there were 6,924, 2,814, and 1,368 incident cases of all-cause dementia, AD, and VaD, respectively, over a median (IQR) follow-up of 13.6 (12.9 to 14.3) years. Participants' characteristics in both cohort studies are shown in Table 1.
Recruitment	The WII study is an ongoing cohort study established in 1985 to 1988 among 10,308 men and women aged 35 to 55, working in twenty civil-service departments in London. Clinical examinations and self-administered questionnaires took place at baseline and subsequently every 4/5 years.<br>The UKB study is a large population-based cohort study on 502,371 participants recruited between 2006 and 2010 among individuals aged 40-69 years registered with the UK National Health Service (NHS). A wide range of information was collected at baseline via touchscreen questionnaires and clinical examinations.
Ethics oversight	The most recent ethics approval for the Whitehall II was from the University College London Hospital Committee on the Ethics of Human Research, reference number 85/0938.<br>This study was performed under generic ethical approval obtained by UK Biobank from the National Health Service National Research Ethics Service (approval letter ref 11/NW/0382, 17 June 2011).

Note that full information on the approval of the study protocol must also be provided in the manuscript.

Field-specific reporting

Please select the one below that is the best fit for your research. If you are not sure, read the appropriate sections before making your selection.

☒ Life sciences ☐ Behavioural & social sciences ☐ Ecological, evolutionary & environmental sciences

For a reference copy of the document with all sections, see nature.com/documents/nr-reporting-summary-flat.pdf

Life sciences study design

All studies must disclose on these points even when the disclosure is negative.

Sample size	Sample size was not decided specifically for the paper as data com from two ongoing cohort studies.<br>Of the 7,870 WII participants at the 1997-1999 wave, the baseline of our analysis, 803 participants with missing data on hearing variables or covariates, and 13 dementia cases before age 65, were excluded, leading to a sample size of 7,054 participants.<br><br>Of the 502,180 UKB participants, 123,398 with missing data on hearing variables or covariates were excluded. In addition, 228 prevalent cases of dementia and 661 cases with onset before age 65, were also excluded. This led to a sample size of 377,893 UKB participants.<br><br>Additionally, no statistical methods were used to pre-determine sample sizes in WII and UKB, but our sample sizes are similar to those reported in previous publications.
Data exclusions	Participants were excluded if they had missing data on the exposure (hearing impairment or haring aid use) and/or covariates. In addition, prevalent dementia at baseline and early onset cases (before age 65 years) were excluded in the main analyses.
Replication	We used data from two cohort studies (Whitehall II and UK Biobank) to examine the robustness of findings in these studies.<br><br>Results for the association between hearing impairment and dementia yielded similar estimates in both cohorts.<br>An increased risk of dementia was observed among hearing aid users in the UK Biobank cohort, but not in the Whitehall II study. Subsequent analyses indicate that this association is more likely attributable to hearing aid use reflecting greater severity of hearing impairment, rather than implying a causal effect.
Randomization	A large set of covariates was used to control for potential confounding factors: age (as time-scale), sex, ethnicity, living alone (marital status in the Whitehall II cohort), education, body mass index and health-related behaviors (smoking, alcohol consumption, fruit and vegetables

consumption, and MET min/week (moderate and vigorous physical activity in the Whitehall II study)), and number of chronic conditions including coronary heart disease, stroke, hypertension, heart failure, diabetes, cancer, chronic kidney disease, chronic obstructive pulmonary disease, liver disease, depression, mental disorders, and arthritis/rheumatoid arthritis.

Blinding

Hearing impairment and hearing aid use were self-reported by the participant in Whitehall II and UK Biobank. Speech-in-noise hearing impairment was assessed using an objective test in UK Biobank. Dementia diagnosis comes from electronic health records, with diagnosis that was blinded to the information on hearing impairment. Statistical analyses conducted by the first author of the paper was not blinded as information on both hearing impairment/hearing aid use and dementia was needed to conduct the analysis.

Reporting for specific materials, systems and methods

We require information from authors about some types of materials, experimental systems and methods used in many studies. Here, indicate whether each material, system or method listed is relevant to your study. If you are not sure if a list item applies to your research, read the appropriate section before selecting a response.

Materials & experimental systems

n/a	Involved in the study
☒	☐ Antibodies
☒	☐ Eukaryotic cell lines
☒	☐ Palaeontology and archaeology
☒	☐ Animals and other organisms
☒	☐ Clinical data
☒	☐ Dual use research of concern
☒	☐ Plants

Methods

n/a	Involved in the study
☒	☐ ChIP-seq
☒	☐ Flow cytometry
☒	☐ MRI-based neuroimaging

Plants

Seed stocks

Report on the source of all seed stocks or other plant material used. If applicable, state the seed stock centre and catalogue number. If plant specimens were collected from the field, describe the collection location, date and sampling procedures.

Novel plant genotypes

Describe the methods by which all novel plant genotypes were produced. This includes those generated by transgenic approaches, gene editing, chemical/radiation-based mutagenesis and hybridization. For transgenic lines, describe the transformation method, the number of independent lines analyzed and the generation upon which experiments were performed. For gene-edited lines, describe the editor used, the endogenous sequence targeted for editing, the targeting guide RNA sequence (if applicable) and how the editor was applied.

Authentication

Describe any authentication procedures for each seed stock used or novel genotype generated. Describe any experiments used to assess the effect of a mutation and, where applicable, how potential secondary effects (e.g. second site T-DNA insertions, mosiacism, off-target gene editing) were examined.
